# The Impact of Plastic Deformation on the Microstructure and Tensile Strength of Haynes 282 Nickel Superalloy Produced by DMLS and Casting

**DOI:** 10.3390/ma15217545

**Published:** 2022-10-27

**Authors:** Ryszard Sitek, Sandra Puchlerska, Ilona Nejman, Kamil Majchrowicz, Zbigniew Pakieła, Krzysztof Żaba, Jarosław Mizera

**Affiliations:** 1 Faculty of Materials Science and Engineering, Warsaw University of Technology, Woloska 141, 02-507 Warsaw, Poland; 2 Faculty of Non-Ferrous Metals, AGH University of Science and Technology, Al. Mickiewicza 30, 30-059 Krakow, Poland

**Keywords:** Haynes 282, superalloy, additive manufacturing, direct metal laser sintering, 3D scanning, microstructure, static tensile test

## Abstract

The article presents the results of research on the influence of plastic deformation on the microstructure and tensile strength of Haynes 282 nickel superalloy produced by direct metal laser sintering (DMLS) and a conventional technique (casting). Samples were tested for dimensional accuracy using a 3D scanner. Then, the samples were subjected to plastic deformation by rolling. The microstructures of the DMLS and the as-cast samples were analysed using a scanning electron microscope. The strength properties of the samples were determined in a static tensile test. Microhardness measurements of the samples were also performed. Based on the analysis of the dimensional accuracy, it was found that the surface quality of the components produced by DMLS is dependent on the input parameters of the 3D printing process. Using the DMLS method, it is possible to produce Haynes 282 with a fine-crystalline microstructure containing dendrites. The fine-crystalline dendritic microstructure and low porosity showed very good tensile strength compared to the as-cast material. It was also found that the increase in the degree of plastic deformation of the as-cast Haynes 282 and the samples produced by the DMLS technique resulted in an increase in the strength of the tested samples, with reduced ductility.

## 1. Introduction

The precipitation-hardened alloy Haynes 282 has excellent properties, such as high strength and creep resistance, due to its γ′ hardening. Additionally, Haynes 282 is highly susceptible to forming, more so than nickel alloys with similar creep strength [1]. Due to this combination of properties, this nickel superalloy is used in the aviation industry for compressors and turbine housings, as well as for exhaust nozzles and diffusers, due to its good weldability [2].

As is well known, the aviation industry is characterised by short-series production. A common method of producing superalloys in aviation is investment casting, which, though widespread, is ineffective in terms of both cost and time. Limitations, such as the development of new wax models and tools for producing them, and the low strength of ceramic molds and core materials [2,3], has led to a search for replacement technologies that would enable more profitable and time-efficient production. Additive manufacturing (AM) may become such a technology. This technique can be defined as a process of combining materials in layers to create physical objects based on data from a 3D model [4]. AM is already used in the aviation industry [5] because of its suitability for short production series, but it can also be used to manufacture a wide range of products.

A number of recent studies on the AM technique have focused on the production of nickel superalloys [4,6,7], including Haynes 282 superalloy [2,8,9,10,11,12,13,14,15,16,17,18].

The authors of the study referenced in [8] conducted research on the PBF processing of Haynes 282. Their work covers PBF process parameter studies, the influence of heating the build plate, and various laser scanning strategies. The samples were characterized by microstructural features (defects and precipitates), hardness, and surface roughness. 

The authors of studies [11,12,13,14,16,17] also conducted research on the Haynes 282 alloy produced using PBF technology. Shaikh et al. [12] focused on the mechanical and microstructural studies of the alloy. They concluded that Haynes 282 manufactured by PBF is a nearly defect-free material, with a density above 99% and virtually no defects such as cracking. Their printed material exhibited a columnar grain structure characteristic of the PBF process, with grain sizes of <30 μm. Robert et al. [13] concluded that the best energy density for the DMLS production of Haynes 282 alloy is 94.5 J/mm^3^. Most of the samples they analysed had cracks, which consisted of several horizontal cracks in the building direction. The cracks were related to the laser scan strategy and may have been caused by hot cracking phenomena. 

Paper [14] looks at the fatigue crack growth behavior of Haynes 282 produced by PBF. Using numerical simulations, the authors showed the influence of grain structure and texture on crack propagation. The numerical results were compared with the experimental results. The analysis showed higher crack propagation along the crack direction in a coarse-grained microstructure and a higher driving force for crack kinking in a fine-grained, more diffusely textured microstructure. This is the effect of grain morphology, texture, hardening rate and yield stress.

Islam et al. [16] demonstrated an experimental and analytical method for quickly defining the additive manufacturing parameters for a new metallic material. Haynes 282 alloy was selected for the research, and 100 samples were examined. The properties of the final products were comparable with the predictions and exhibited high ductility and strength.

In the study referenced in [17], correlations between the microstructure, mechanical properties and post-processing of Haynes 282 were studied. The γ-phase was observed only after the second heat treatment, whereas HIP precipitates the γ-phase in a single step. The studies conducted in work [11] also concerned the effect of Haynes 282 heat treatment, in particular the effects of lower heat treatment temperatures on the microstructure and tensile properties of Haynes 282 produced by PBF. It was shown that a solution temperature range of 1062–1146 °C could reduce the dendritic microstructure, resulting in a similar distribution of grain boundary carbides. 

Studies [9,10,15] relate to other methods of additively producing Haynes 282. In study [9], samples of Haynes 282 alloy were fabricated by liquid deposition modeling (LMD). The effect of several heat treatments was studied to obtain new, improved properties. Unocic et al. [10] studied the electron beam melting (EBM) process applied to Haynes 282. The authors modified the parameters of the printing process in order to select those that were optimal for the production of Haynes 282 alloy. The key parameters for reducing porosity and cracking were hatch spacing, beam focus, beam current, and scanning strategy. Fernandez-Zelaia et al. [15] utilized EBM to produce the Haynes 282 alloy with a varying mesoscale structure. The authors concluded that the crack growth rate is dependent on the microstructural heterogeneity.

In the literature, studies can be found on other nickel superalloys produced by the PBF method in which the structure of the manufactured materials was characterized by above-average properties compared with alloys produced by conventional methods. Using the PBF method, the authors of study [19] obtained an Inconel 718 superalloy whose properties were superior to those obtained using the other two methods (SPS, casting) and which had the highest density and hardness. As a result, an intact microstructure showing lower segregation and precipitation in the matrix than the as-cast material was obtained. Some researchers [20] suggest that the PBF method can allow materials with a specific microstructure to be obtained, with higher product properties than those obtained using conventional methods, e.g., casting. This is prompting researchers to experiment with the plastic deformation of materials produced using AM methods.

Although both cast and DMLS Haynes 282 nickel superalloy are studied extensively, most published studies focus on conventional heat treatment and post-processing as described above. Moreover, no study has yet compared the microstructure and mechanical properties of the as-cast and deformed Haynes 282 superalloy. Apart from the obvious uses of the Haynes 282 alloy, the search for new applications through plastic deformation leading to increased strength and deliberate anisotropy can be a very interesting area of research.

In this study, samples made using the DMLS method and by casting were tested. Three-dimensional scanning of the products was performed to compare how their geometries deviated from the nominal values. The quality of the products was assessed, and the accuracy of the two methods compared. In the next stage of the research, the samples were subjected to plastic deformation by rolling with different degrees of deformation: 20%, 40% and 60%. The microstructures of the DMLS and as-cast samples were then analysed, and static tensile tests and microhardness measurements were performed. The effect of deformation on the structure and mechanical properties of the DMLS and as-cast samples was examined.

## 2. Materials and Methods

In the first step, two samples were produced from the Haynes 282 nickel superalloy. The chemical composition of the alloy is shown in Table 1.

**Figure 1:**
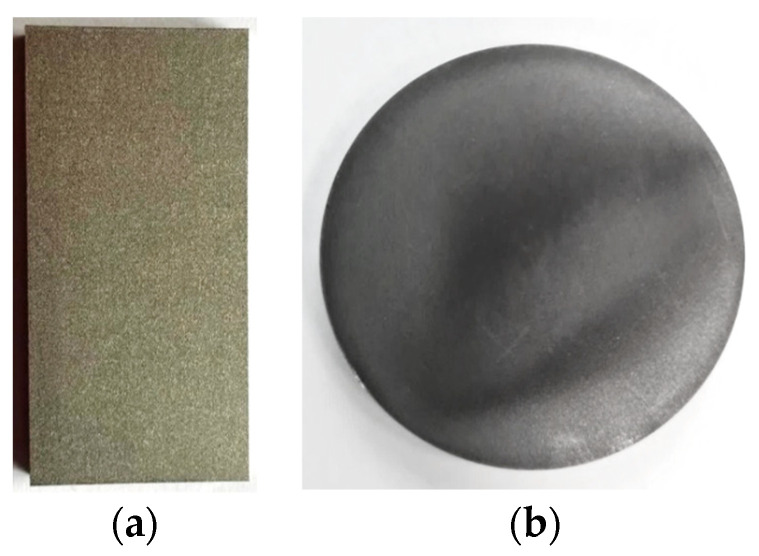
(**a**) DMLS sample; (**b**) as-cast sample.

The thickness of the samples was 5 mm. The DMLS sample was produced on an EOS M 100 device using an EOS M100 3D printer (EOS GmbH Electro Optical Systems, Krailling, Germany) operating in DMLS technology. A test specimen in the shape of a cuboid with dimensions of 50 mm × 70 mm × 5 mm was produced using the following parameters: layer thickness = 20 µm, laser power = 90 W, scanning speed = 800 mm/s, volumetric energy density = 94 J/mm^3^. A scanning strategy was used in which the scanning lines relative to the previous layer were rotated through an angle of 67° to reduce anisotropy. The DirectBase S15 platform was heated to a temperature of 80 °C during the process. The oxygen content in the working chamber was <0.1% due to the use of argon as a protective gas. The as-cast sample was tested in the annealed condition. The test samples are shown in Figure 1.

In the first stage of the research, 3D scanning of the produced samples was performed. A Gom Atos Core 200 3D scanner (Carl Zeiss GOM Metrology GmbH, Braunschweig, Germany) was used for this purpose. The accuracy of the device was 0.018 mm. No scanning spray was used for the scanning. Gom Inspect 2018 software was used to analyse the scans; they were compared with the CAD files in order to determine the geometric deviation. Surface topography was determined using the appropriate functions of the Gom Inspect software. 

In the next stage, samples for plastic deformation were prepared. The samples were cut from the base material with an EDM machine, taking into account the building direction of the DMLS sample. The plastic deformation was carried out using a quarto reversible rolling mill with a roll diameter of 60 mm and a speed of 20 rpm. The degrees of deformation were 20%, 40% and 60%. The plan of the deformation experiment is presented in Table 2. 

Afterwards, samples for the microstructure studies were prepared from the base materials and after plastic deformation. The research was carried out on a Hitachi SU-70 (Tokyo, Japan) scanning electron microscope. The observations were made at a voltage of 5 kV with a magnification of 1000×. A point analysis of the chemical composition was carried out. 

Microhardness tests HV 0.05 were performed on the samples prepared for the microstructure studies. The research was carried out using a Shimadzu HMV-2T E (Kyoto, Japan) hardness tester. Six measurements were made from each sample. 

**Figure 2:**
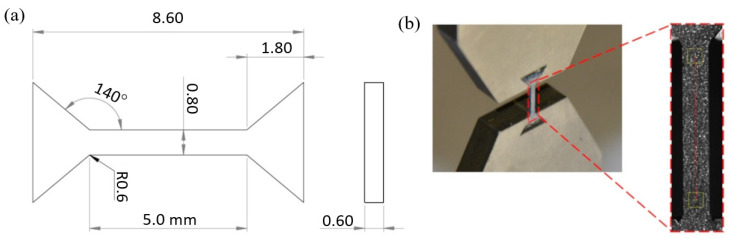
(**a**) Technical drawing of miniaturised tensile sample, and (**b**) its actual view with a schematically presented digital image of its surface used for DIC analysis.

The as-cast and additively manufactured samples following plastic deformation were also characterized in terms of their mechanical properties as measured in uniaxial tensile tests. The miniaturized tensile specimens with a gauge length of 5 mm and a cross-section of 0.8 mm × 0.6 mm (as presented in Figure 2 and described in more detail in [22,23]) were cut using an electrical discharge machine (EDM) in the rolling direction. The tensile experiments were performed at an initial strain rate of 10^−3^ s^−1^ using a Zwick/Roell Z005 (Ulm, Germany) static testing machine with a loading capacity of 5 kN and a digital image correlation (DIC) system for the strain measurements. Digital images of the sample surface (Figure 2b) were registered at a frequency of 4 Hz using an AVT Pike F-505B ASG (Allied Vision Technologies GmbH, Stadtroda, Germany) CCD camera and processed using VIC-2D Correlated Solutions (Correlated Solutions Inc, Irmo, SC, USA) software. Each material was represented by at least three test specimens. The 0.2% offset yield strength (YS), ultimate tensile strength (UTS), and elongation to failure (A) were calculated from the stress–strain curves obtained, following the ISO 6892-1 standard procedure.

## 3. Results and Discussion

**Figure 3:**
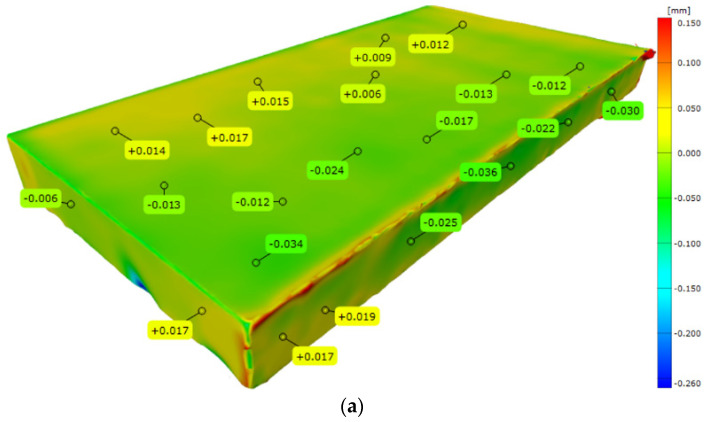

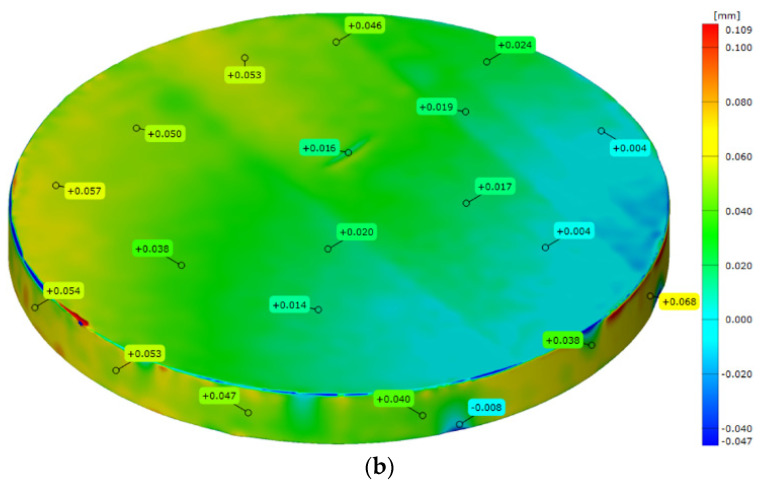
Deviation maps for scans superimposed on nominal CAD models: (**a**) DMLS sample; (**b**) as-cast sample.

**Figure 4:**
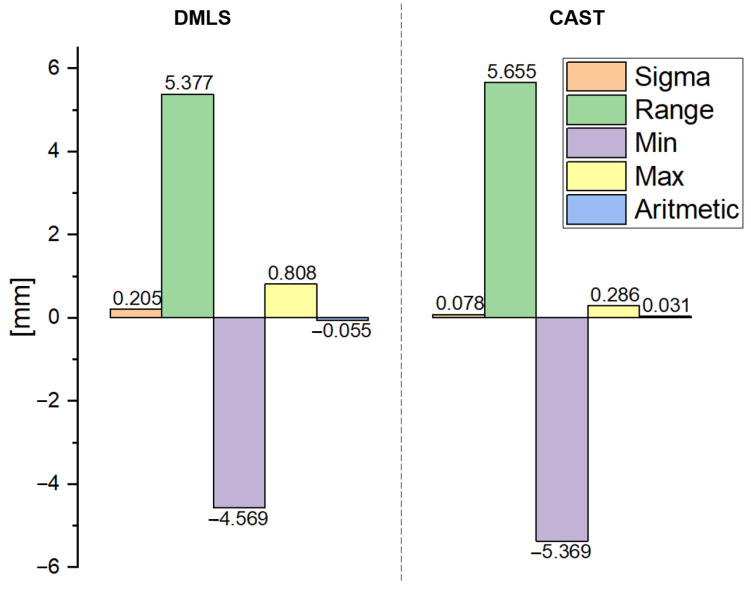
Statistical data generated in the Gom Inspect software for the DMLS and as-cast samples.

**Figure 5:**
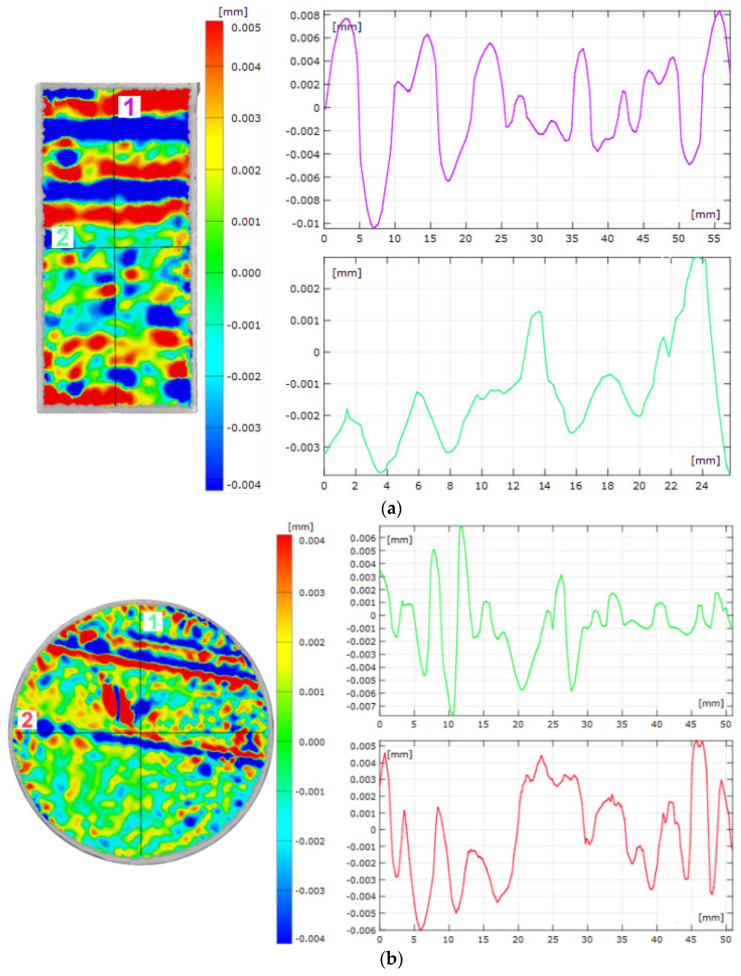
Surface topography: (**a**) DMLS sample; (**b**) as-cast sample. Number 1 is the measurement perpendicular to the sample building direction, while number 2 is the measurement taken parallel. The same measurement methodology was used for the cast sample.

Figure 3 shows the results of a comparison of the 3D scans of the DMLS and as-cast samples with the nominal CAD models. The statistics generated in the Gom Inspect software are shown in Figure 4. Figure 5 shows the surface topography analysis of the DMLS and as-cast samples.

Based on these results (Figure 3 and Figure 4), it can be concluded that the surface analysis results for the DMLS sample and for the as-cast sample do not differ significantly. The scatter of the results for both samples was similar (for the DMLS sample = 5.377 and for the as-cast sample = 5.655). The standard deviation of the results shows that the scatter of the results around the mean was larger for the DMLS sample (0.205) than for the as-cast sample (0.078). As indicated in the literature [6], the surface quality of 3D-printed metallic components depends on many factors, including the input parameters related to the raw material, the design, the process parameters, and finally post-processing. A final surface treatment such as grinding, post HIP or chemical polishing is recommended for 3D-printed metallic elements [24].

Many parameters of the 3D printing process determine the AM product topography. These parameters are related to the quality of the batch powder, the spatial design of the part, the process parameters and the final treatment [6]. Of the process parameters, the most important are the laser beam guidance strategy, the laser spot diameter, the laser power, the laser feed, and the layer thickness. The surface roughness of AM objects is a critical element in process design. High quality is required for components used in aviation, so it is extremely important to plan appropriate post-treatment for products. 

Elements manufactured with the AM for high-quality applications require an average surface roughness below 1 mm [25]. For the samples analysed during our work (Figure 5), the Ra_1C_ parameter for the DMLS sample was 4.73 µm, whereas the Ra_2C_ was 1.87 µm. The Ra_1DMLS_ parameter for the as-cast sample was 1.97 µm, whereas the Ra_2DMLS_ was 2.46 µm. The results demonstrate the high surface quality of the manufactured parts. The Ra_1DMLS_ parameter was more than 2.5 times higher than the Ra_2DMLS_ parameter, which was the correct tendency given that the Ra_1DMLS_ was analysed perpendicular to the building direction. The authors of [26] measured roughness with standard devices for this purpose, i.e., with profilometers or using SEM. In this study, an unconventional method of measuring roughness is proposed.

**Figure 6:**
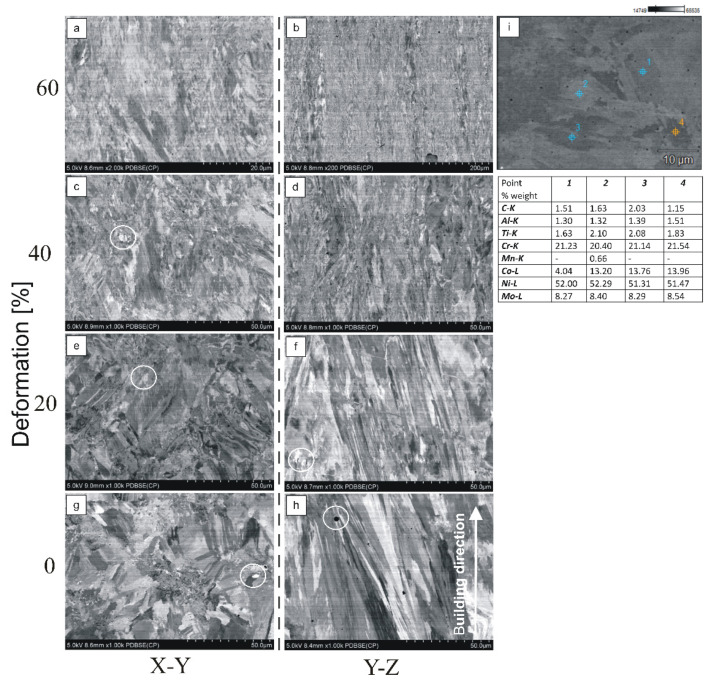
Microstructure of DMLS samples depending on the construction direction and point analysis of the chemical composition. (**a**) 60% deformation, cross section; (**b**) 60% deformation, longitudinal section; (**c**) 40% deformation, cross section; (**d**) 40% deformation, longitudinal section; (**e**) 20% deformation, cross section; (**f**) 20% deformation, longitudinal section; (**g**) initial state, cross section; (**h**) initial state, longitudinal section; (**i**) point analysis of the chemical composition.

Based on the microstructure analysis, no cracks or other material defects were observed. Porosity (Figure 6h) with a maximum diameter of approx. 2 µm was observed. The point analysis of the chemical composition (Figure 6i) showed that the alloy is rich in nickel and chromium. Figure 6g–h shows the dendritic structure in the initial state. The microstructure was columnar-grained. It can be observed that the dendrites have a similar growth direction to the sample building direction and grow along it (Figure 6h), which is a characteristic phenomenon for nickel superalloys produced by AM techniques [9,12,27]. Compositional contrast from the back-scattered electron (BSE) detector shows bright spots marked with white circles (Figure 6c,e,g). This represents a segregation of heavy element compounds in the interdendritic areas. As deformation increases, the grains elongate due to a deformation of the dendritic structure. In the strongly deformed structure (Figure 6c) the matrix grain boundaries are not visible; however, the bright zones in the structure are still clearly noticeable. This proves that the grains deformed more easily than the precipitation. Similar observations have been made by other authors [12], using a laser power of 400 W and an EOS 3D printer. The EDX analysis maps presented in that work highlighted the segregation of C, Ti, and Mo to the interdendritic regions. The dendrite cores were conversely rich in Cr, Ni, and Co, and Al was found to be evenly distributed throughout the solidification microstructure. 

**Figure 7:**
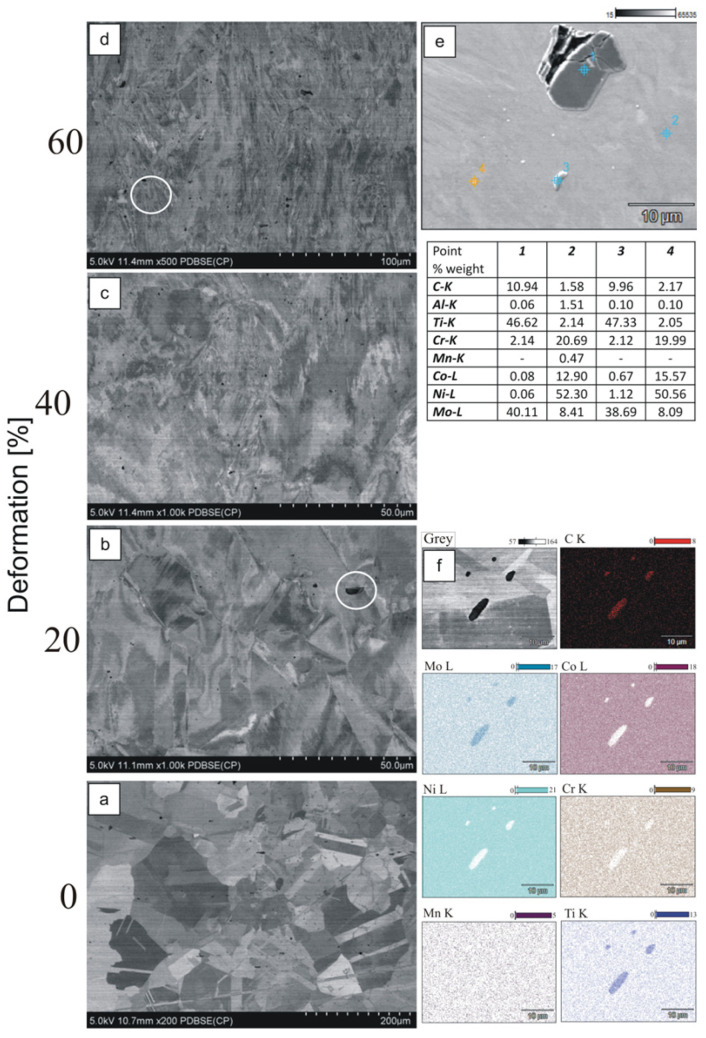
Microstructure of as-cast samples and point analysis of the chemical composition (60% deformation). (**a**) base as-cast sample; (**b**) 20% deformation; (**c**) 40% deformation; (**d**) 60% deformation (**e**) point analysis of the chemical composition; (**f**) chemical analysis composition. (mapping).

The microstructure of the base as-cast sample is characterized by large grains (Figure 7a) with clearly visible grain boundaries. After deformation, the grains are elongated, and their boundaries are no longer clear (Figure 7b–d). In the microstructure there is also randomly distributed MC, such as (Ti, Mo)C precipitates, preferentially in the interdendritic regions and grain boundaries (Figure 7b–d). A point analysis of the precipitations and mapping (Figure 7e–f) showed that the carbides are rich in titanium, molybdenum (Ti, Mo-MC), and chromium (M_23_C_6_). Based on the microstructure photography, it can be concluded that the precipitations are brittle following intensive plastic deformation. Vacuum casting and deoxidation processes are advisable to improve the mechanical properties of Haynes 282 alloy. These treatments can help inhibit the formation of large Ti-rich MX. It is important to reduce the amount of Ti-rich CX type carbides because Ti is the component of the main strengthening phase γ′ of the alloy [28]. 

**Figure 8:**
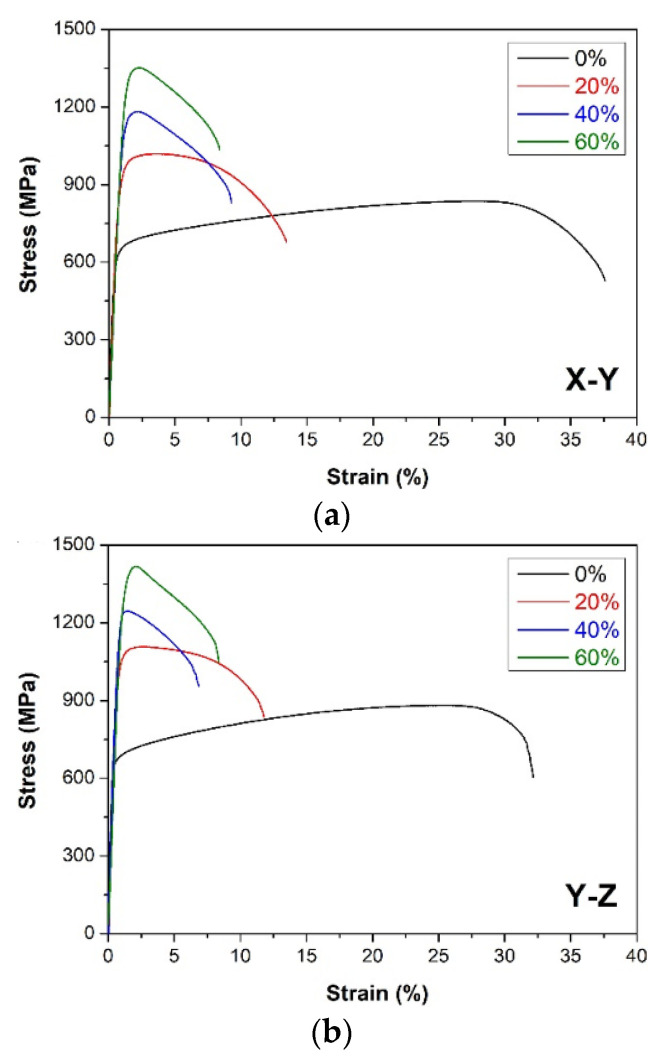

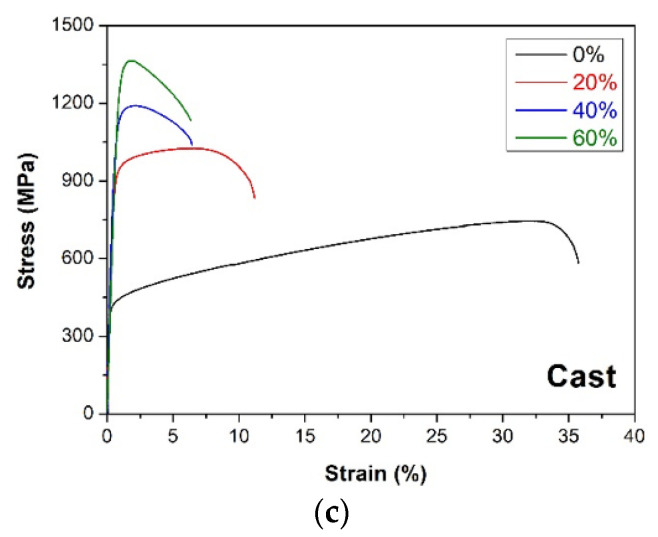
Stress–strain curves of (**a**) DMLS samples of Haynes 282 built in the X-Y direction; (**b**) DMLS samples of Haynes 282 built in the Y-Z direction; (**c**) as-cast Haynes 282. The black curves represent the results for the samples in the initial state, the red curves are for the samples after 20% deformation, the blue curves for the samples after 40% deformation, and the green curves for the samples after 60% deformation.

Representative stress–strain curves for the DMLS and as-cast Haynes 282 after further plastic deformation are presented in Figure 8. The mechanical properties calculated are summarized in Table 3. The as-cast Haynes 282 exhibited a YS of 421 ± 8 MPa, UTS = 742 ± 28 MPa, and A = 35.0 ± 1.7%, whereas the additively manufactured samples showed a much higher YS (594 ± 8 and 656 ± 18 MPa for X-Z and Y-Z orientation, respectively) and UTS (835 ± 7 and 879 ± 26 MPa), with a similar elongation to failure (A = 36.9 ± 3.4 and 31.7 ± 3.1%). The results obtained for the DMLS Haynes 282 are comparable with other literature data (YS = 633 MPa, A = 31.5% [9]). Further plastic deformation of both the as-cast and the DMLS Haynes 282 resulted in a gradual improvement in strength, with an increasing deformation degree at the expense of gradually reduced ductility (Table 3). It should also be noted that the Y-Z DMLS specimens exhibited higher strength and slightly lower ductility than their counterparts with an X-Y orientation. These differences resulted from the microstructure anisotropy of the DMLS Haynes 282; that is, the columnar grains growing in the building direction (*Z*-axis) provided enhanced strength in the Y-Z orientation, whereas the more homogeneous microstructure in the X-Y plane ensured slightly higher ductility of the X-Y specimens. Finally, a comparison of the mechanical properties of the as-cast and DMLS samples showed that the as-cast Haynes 282 was more prone to strengthening by plastic deformation than the DMLS samples. Both the YS and UTS improved much more in the case of as-cast Haynes 282 (by approximately ΔYS = 840 MPa and ΔUTS = 670 MPa for the highest deformation degree). The plastic deformation of the DMLS samples was not so effective (ΔYS = 629 MPa and ΔUTS = 510 MPa for X-Y and ΔYS = 609 MPa and ΔUTS = 539 MPa for Y-Z). This resulted from the significantly improved mechanical properties of the DMLS samples directly after the DMLS processing and because of the much higher strain hardening capability shown by the as-cast Haynes 282, i.e., the difference between the YS and UTS (UTS-YS = 321 MPa) was higher than in the DMLS samples (241 and 223 MPa for X-Y and Y-Z orientation, respectively). Nevertheless, the combination of the highest strength and ductility was still obtained for the DMLS sample with a Y-Z building orientation.

**Figure 9:**
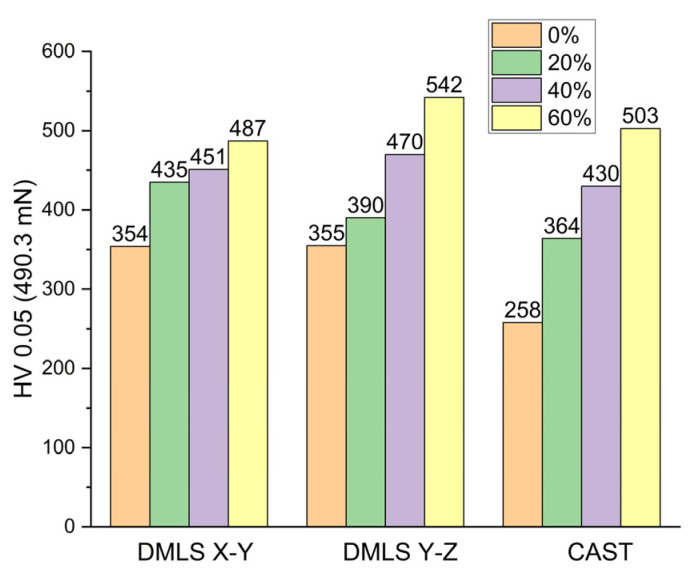
Microhardness of DMLS and as-cast samples.

Based on the results of the microhardness tests (Figure 9), it was found that, in the case of DMLS samples in the initial state, the direction of building did not affect the hardness (HV 0.05 = 355 for X-Y and HV 0.05 = 354 for Y-Z). The 20% deformed DMLS sample built in the X-Y direction had an average HV value higher (HV 0.05 = 435) than the DMLS sample built in the Y-Z direction (HV 0.05 = 390). Then, as the deformation degree increased (to 40% and 60%), the hardness of the sample built in the Y-Z direction proved to be higher than that of the sample built in the X-Y direction (by 21 units at 40% strain and 55 units at 60% strain). These results are consistent with the tensile test results. The samples built in the Y-Z direction were characterized by higher strength, whereas the samples built in the X-Y direction were characterized by higher ductility. The hardness of the cast material was lower for the base sample and for the deformed samples than for the DMLS samples. The results of the microhardness tests are consistent with the results of the mechanical properties tests. The Haynes 282 cast material was more susceptible to deformation strengthening than the DMLS material. This is proved by the intense increase in the microhardness of the cast sample, along which the sample deformation was increased.

## 4. Conclusions

- Based on the results of the 3D scanning, it can be concluded that the surface quality of DMLS and as-cast samples in the base state are at a similar level.
- The quality of the surfaces of 3D printed materials depends on factors such as the parameters of the raw material (the metallic powder), the design process, the 3D printing parameters, and the post-processing.
- During the microscopic examinations of the DMLS samples, no cracks were found. There was slight porosity in the structure. In the structure of the cast samples, precipitations of MX-type carbides were observed.
- Haynes 282 nickel superalloy with a dendritic microstructure can be produced by the DMLS method. The fine crystalline dendritic microstructure and low porosity showed very good tensile strength compared with the cast material.
- An increase in the degree of plastic deformation of the Haynes 282 materials produced by DMLS and by casting resulted in an increase in the tensile strength of the tested samples, along with a reduction in ductility. The results of the microhardness tests were consistent with the results of the mechanical properties tests.

## Figures and Tables

**Table 1:** Chemical composition [% wt.] of the Haynes 282 alloy [21].

Ni	Cr	Co	Mo	Ti	Al	Fe	Mg	Si	C	B
<57	20	10	8.5	1.2	1.5	<1.5	0.3	<0.15	0.06	0.005

**Table 2:** Deformation plan of the DMLS and as-cast sample.

No	Manufacturing Method	Orientation	Deformation [%]
1	DMLS	X-Y	0
2	DMLS	X-Y	20
3	DMLS	X-Y	40
4	DMLS	X-Y	60
5	DMLS	Y-Z	0
6	DMLS	Y-Z	20
7	DMLS	Y-Z	40
8	DMLS	Y-Z	60
9	Cast	-	0
10	Cast	-	20
11	Cast	-	40
12	Cast	-	60

**Table 3:** Mechanical properties of DMLS and as-cast Haynes 282 after plastic deformation.

Manufacturing Method	Building Orientation	Deformation Degree (%)	YS (MPa)	UTS (MPa)	A (%)
DMLS	X-Y	0	594 ± 8	835 ± 7	36.9 ± 3.4
		20	863 ± 16	1019 ± 24	13.0 ± 2.4
		40	1036 ± 29	1141 ± 18	8.6 ± 1.4
		60	1223 ± 18	1345 ± 9	7.7 ± 0.2
	Y-Z	0	656 ± 18	879 ± 26	31.7 ± 3.1
		20	968 ± 22	1094 ± 12	11.5 ± 1.1
		40	1074 ± 18	1235 ± 13	5.7 ± 0.9
		60	1265 ± 42	1418 ± 48	6.7 ± 1.4
Casting	-	0	421 ± 8	742 ± 28	35.0 ± 1.7
		20	902 ± 21	1022 ± 19	10.7 ± 0.3
		40	1043 ± 9	1164 ± 19	7.1 ± 1.1
		60	1261 ± 42	1412 ± 53	6.2 ± 0.7

## Data Availability

Data are available with the first author and can be shared with anyone upon reasonable request.
